# High-yield cell-free synthesis of human EGFR by IRES-mediated protein translation in a continuous exchange cell-free reaction format

**DOI:** 10.1038/srep30399

**Published:** 2016-07-26

**Authors:** Robert B. Quast, Andrei Sonnabend, Marlitt Stech, Doreen A. Wüstenhagen, Stefan Kubick

**Affiliations:** 1 Fraunhofer Institute for Cell Therapy and Immunology (IZI), Branch Bioanalytics and Bioprocesses (IZI-BB), Am Mühlenberg 13, D-14476 Potsdam, Germany

## Abstract

Cell-free protein synthesis systems derived from eukaryotic sources often provide comparatively low amounts of several μg per ml of *de novo* synthesized membrane protein. In order to overcome this, we herein demonstrate the high-yield cell-free synthesis of the human EGFR in a microsome-containing system derived from cultured *Sf*21 cells. Yields were increased more than 100-fold to more than 285 μg/ml by combination of IRES-mediated protein translation with a continuous exchange cell-free reaction format that allowed for prolonged reaction lifetimes exceeding 24 hours. In addition, an orthogonal cell-free translation system is presented that enabled the site-directed incorporation of p-Azido-L-phenylalanine by amber suppression. Functionality of cell-free synthesized receptor molecules is demonstrated by investigation of autophosphorylation activity in the absence of ligand and interaction with the cell-free synthesized adapter molecule Grb2.

Membrane proteins are among the most abundant classes of proteins and due to their importance in the context of cell progression and viability, malfunction has in many cases been found to correlate with disease^1^,^2^. A prominent and well-studied example is the human epidermal growth factor receptor (EGFR), a type I single transmembrane-spanning receptor tyrosine kinase involved in cell differentiation and proliferation^3^. Overexpression and mutations that result in aberrant receptor signaling have been found to play an important role in the development of different carcinoma types^4^. Although the mechanisms of EGFR activation and signal propagation have been extensively studied, controversial data suggests a complex regulatory interplay that remains to be further elucidated in order to provide sufficient information for the discovery of novel pharmaceutical targets (for a more detailed overview please refer to^5^,^6^,^7^).

The inherent nature of integral membrane proteins, in particular their partly hydrophobic character and correspondingly concerted folding in association with the membranous lipid bilayer, complicates their production and purification in a functional form^8^. Moreover, their overexpression can have a significant impact on the host’s vitality limiting overall yields obtainable^9^. In this context, cell-free protein synthesis (CFPS) can be considered a valuable alternative. Lacking the constraints accompanied by the cellular plasma membrane, protein synthesis can be focused on the production of the target protein and conditions can be directly adapted to account for individual requirements. Moreover, the generation of artificial hydrophobic environments by addition of supplements has proven useful for the production of membrane proteins^10^,^11^. Nevertheless, these artificial environments only mimic the native surrounding membrane proteins encounter in the cellular context. As many membrane proteins are initially synthesized into the membranes of the endoplasmic reticulum (ER) before being transported to their final destinations, we established a cell-free protein synthesis system derived from cultured *Spodoptera frugiperda* 21 (*Sf*21) cells, which comprises endogenous ER membranes^12^. These endogenous microsomal structures have been demonstrated to be translocationally active, thereby supporting the signal peptide mediated translocation of proteins across the membrane, and further provide functions such as signal peptide cleavage, N-glycosylation and lipid modifications^13^,^14^,^15^,^16^. Moreover, proper folding of eukaryotic proteins is most likely facilitated by the inherent nature of the extract source.

Although enabling the synthesis of difficult-to-express proteins such as membrane proteins, a main limitation of the cell-free *Sf*21 system is the comparatively low protein yield that can be obtained in the standard batch formatted reaction^17^. Therefore, we demonstrated the possibility of increasing the reaction lifetime using a commercially available dialysis chamber composed of a reaction compartment and a feeding compartment^18^. This so-called continuous exchange cell-free (CECF) reaction format provided accumulation of different target proteins in the reaction chamber, thereby increasing total protein yields obtained from a single cell-free reaction. In a different study, we investigated on the use of viral internal ribosome entry sites (IRES) in order to increase the productivity of different eukaryotic cell-free systems, including the *Sf*21 system, in batch formatted reactions^19^. As the availability of eukaryotic initiation factors had been previously demonstrated to limit protein yields in a cell-free HeLa system^20^, it was not surprising that the use of the cricket paralysis virus (CrPV) IRES, which promotes protein translation independent of initiation factors^21^, increased the productivity of cell-free protein synthesis in the *Sf*21 system.

In this study, we correspondingly combined the IRES-mediated cell-free synthesis approach with the CECF format in order to investigate their impact on yields of the integral membrane protein EGFR in the cell-free microsome-containing *Sf*21 system. Additionally, the previously described orthogonal cell-free translation system (OcfTS), composed of a tyrosyl-tRNA synthetase mutant (eAzFRS) and a natural amber suppressor tRNA_CUA_^17^, was utilized to incorporate p-Azido-L-phenyalanine (AzF) into the EGFR by amber suppression under the novel synthesis conditions. Finally, the functionality of cell-free synthesized wild type EGFR and EGFR with incorporated AzF was addressed based on phosphorylation of selected tyrosine residues in the receptor’s C-terminal tail and interaction with the cell-free synthesized adapter protein growth factor receptor bound protein 2 (Grb2).

## Results

### IRES-mediated cell-free synthesis of human EGFR in CECF reaction format

**Figure 1:**
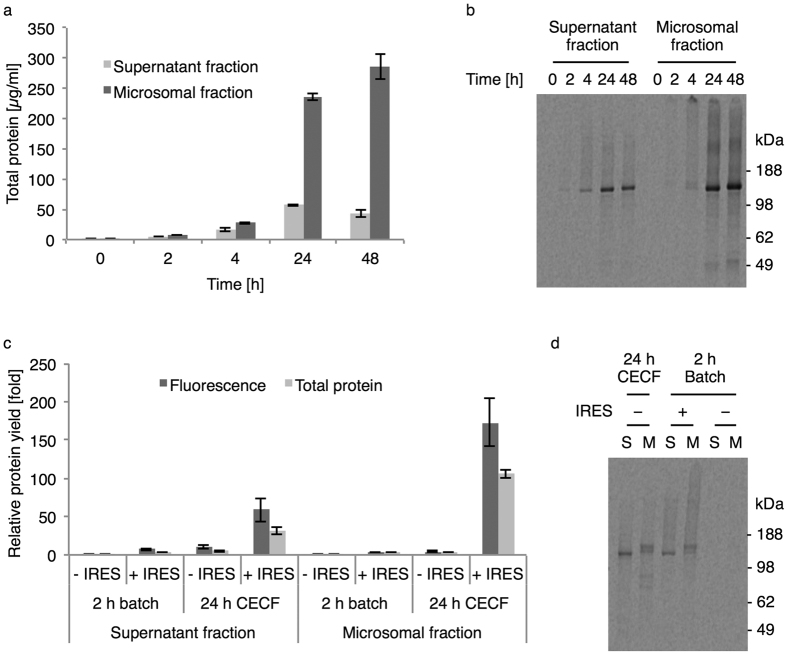
IRES-mediated cell-free synthesis of EGFR-eYFP in the CECF reaction format. (**a**) Total protein yields of EGFR-eYFP at different time points. (**b**) Autoradiography of corresponding samples after electrophoretic separation. (**c**) Relative yields of total protein and eYFP fluorescence obtained using the standard (−IRES) and IRES template (+CECF) in the batch or CECF format in relation to the standard reaction (−IRES, 2 h batch). (**d**) Autoradiography of corresponding samples from supernatant (S) and microsomal fractions (M) after electrophoretic separation. Error bars represent the standard deviation of triplicate analysis. The autoradiograms (**b**,**d**) have been adapted in contrast, brightness and sharpness for better visibility. The original image can be found in supplementary Fig. 1.

We previously demonstrated the capability of our microsome-containing cell-free system based on cultured *Sf*21 cells to synthesize the glycosylated human EGFR in full-length, but unfortunately yields were limited to only several μg per ml^17^. As protein translation independent of eukaryotic initiation factors has been shown to provide increased synthesis rates in different eukaryotic cell-free systems including the *Sf*21 system^19^, we implemented the CrPV-IRES into the corresponding vector upstream of the EGFR gene. Moreover, we have been able to show that reaction lifetimes are prolonged using the cell-free *Sf*21 system in a CECF reaction format^18^. Therefore, we carried out the IRES-mediated translation in the coupled transcription/translation mode under adapted conditions in a commercially available CECF device and analyzed total protein yields as well as fluorescence of an N-terminal enhanced yellow fluorescent protein (eYFP) fusion in the supernatant and microsomal fractions obtained by centrifugation. Although cell-free protein synthesis proceeded up to 48 hours the highest productivity was found to be in the range of 24 hours yielding 57 μg/ml in the supernatant fraction and 237 μg/ml in the microsomal fraction (Fig. 1a and Table 1). In comparison to previously described standard conditions of initiation-dependent cell-free synthesis from templates without IRES in the batch mode^17^, total protein yields increased equally by 3.8-fold using the IRES template (2 h) or using the template without IRES in the CECF format (24 h) in the microsomal fraction (Fig. 1c, Total protein). The combination of IRES-mediated CFPS carried out in the CECF format resulted in more than a 100-fold increase in 24 hours. Interestingly, the increase in fluorescence of the eYFP fusion was even more pronounced by the prolonged reaction time as only a 3.1-fold increase was observed in the microsomal fraction using the IRES in the batch mode but a 4.3-fold increase was found without IRES in the CECF mode and more than 170-fold in combination after 24 hours (Fig. 1c, Fluorescence). Incubation of the IRES-mediated cell-free reaction in the CECF format for 48 hours yielded more than 285 μg/ml total protein representing an increase of 128.2-fold compared to the standard reaction and the eYFP fluorescence increased by 232.2-fold. Autoradiography revealed synthesis of the EGFR-eYFP fusion protein from reactions with IRES in the CECF (Fig. 1b) as well as batch mode and without IRES in the CECF mode but no protein was detected in the standard reaction under the applied labeling conditions (Fig. 1d). In accordance to earlier findings this confirmed the capability of the *Sf*21 microsomes to provide N-linked glycosylation of cell-free synthesized proteins^17^,^19^, as autoradiography from microsomal fractions of reactions carried out with the IRES template in the batch mode and without IRES in the CECF mode exhibited a shift in the migration pattern corresponding to a higher molecular weight compared to the distance found in the supernatant fractions (Fig. 1d). In contrast, the EGFR-eYFP synthesized from the IRES template in the CECF mode was migrating as a single well-defined band after 24 as well as 48 hours, being of equal size independent from which fraction it originated but in the microsomal fraction after 4 hours, a faint band was visible at a corresponding higher molecular weight (Fig. 1b). Additionally, a faint side product at roughly 49 kDa was detected after 24 and 48 hours in the microsomal fraction.

### Incorporation of AzF by an orthogonal tRNA/synthetase pair in the CECF format

We previously described a novel OcfTS based on the microsome-containing *Sf*21 system presented here that provided the co-translational incorporation of AzF into glycosylated proteins and membrane proteins by amber suppression^17^,^22^. The incorporation was facilitated using a mutant *Escherichia coli* (*E. coli*) tyrosyl-tRNA synthetase, which was first described by Chin *et al*.^23^, in combination with a natural *E. coli* amber suppressor tRNA_CUA_. Due to an unsatisfying specificity of the synthetase for the suppressor tRNA_CUA_, which resulted in misincorporation of AzF in response to codons other than the amber codon, we further implemented an arginine 265 mutation into the synthetase according to Takimoto *et al*.^24^. As expected, this mutation within the anticodon recognition site of the synthetase increased the efficiency of AzF incorporation but also improved the specificity of incorporation (unpublished data).

**Figure 2:**
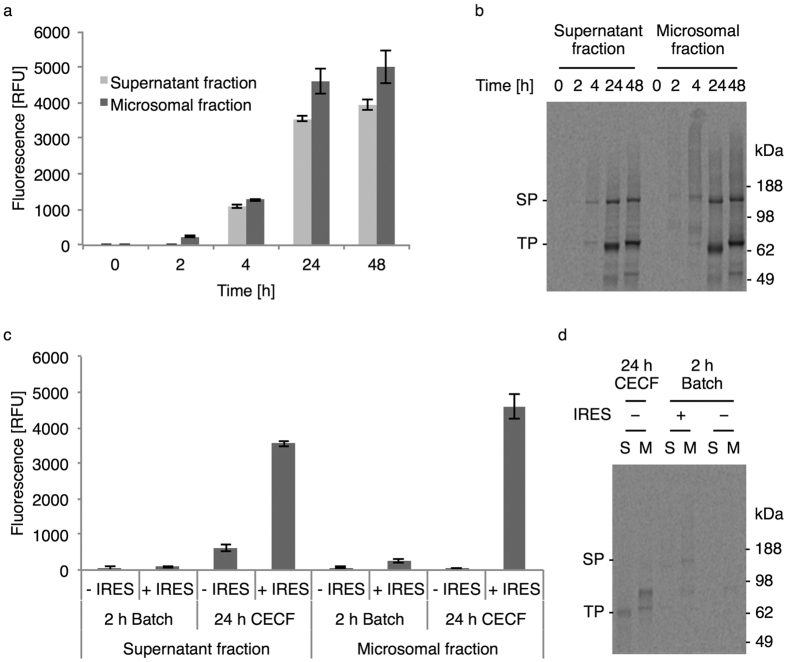
IRES-mediated cell-free synthesis of EGFR-eYFP-AzF687 by amber suppression using an orthogonal tRNA/synthetase pair in the CECF reaction format. (**a**) Relative eYFP fluorescence of the full-length suppression product at different time points. (**b**) Autoradiography of corresponding samples after electrophoretic separation. (**c**) Relative eYFP fluorescence of the full-length suppression product obtained using the standard (−IRES) and IRES template (+IRES) in the batch or CECF format. (**d**) Autoradiography of corresponding samples from supernatant (S) and microsomal fractions (M) after electrophoretic separation. The full-length suppression product (SP) and the termination product (TP) are indicated. Error bars represent the standard deviation of triplicate analysis. The autoradiograms (**b**,**d**) have been adapted in contrast, brightness and sharpness for better visibility. The original image can be found in supplementary Fig. 1.

In order to investigate the influence of IRES-mediated cell-free synthesis in the CECF format on yields of the full-length suppression product, corresponding amber mutants were generated to incorporate AzF at the position corresponding to amino acid 687 within the EGFR. Detection of the full-length suppression product was achieved by monitoring the fluorescence of the C-terminal eYFP fusion, which was only synthesized upon successful suppression of the internal amber codon. In accordance with the synthesis of the wild type EGFR-eYFP the highest productivity was found to be within 24 hours of reaction time using the IRES template in the CECF mode but with only slightly more suppression product in the microsomal fraction (Fig. 2a). Increased incubation up to 48 hours did not result in a further increase of full-length suppression product. Autoradiography confirmed the synthesis of the full-length suppression product in both fractions (Fig. 2b, SP) but further revealed a significant translation termination at the internal amber codon (Fig. 2b, TP). Moreover, the side product observed when synthesizing the wild type EGFR-eYFP was also detected at approximately 49 kDa. Comparison of IRES-mediated synthesis with IRES-independent synthesis in the different reaction modes based on the eYFP fluorescence, clearly reflected the benefit of combining IRES-mediated synthesis with the CECF format as hardly any full-length suppression product was detected from the other reactions (Fig. 2c). Unexpectedly, the supernatant fraction from the CECF reaction using the template without IRES clearly exhibited fluorescence even though no full-length suppression product was visible in the autoradiogram. It should be noted that for all the different reaction conditions a corresponding control reaction without gene template was performed and the measured background was subtracted from recorded eYFP intensities. In accordance with the observation from the synthesis of wild type EGFR-eYFP, differences in the migration pattern of the suppression as well as termination products between the supernatant and microsomal fractions reflected the occurrence of N-glycosylation. Estimation of total yields, based on the eYFP fluorescence of wild-type EGFR-eYFP and suppression product and the total yields of the isotopically labeled wild type, revealed synthesis of around 17 μg/ml of full-length suppression product when combining IRES-mediated synthesis with the CECF reaction format. The suppression efficiency calculated in relation to the eYFP fluorescence of the corresponding wild type synthesis was estimated to be 7%.

### Autophosphorylation activity of cell-free synthesized EGFR

**Figure 3:**
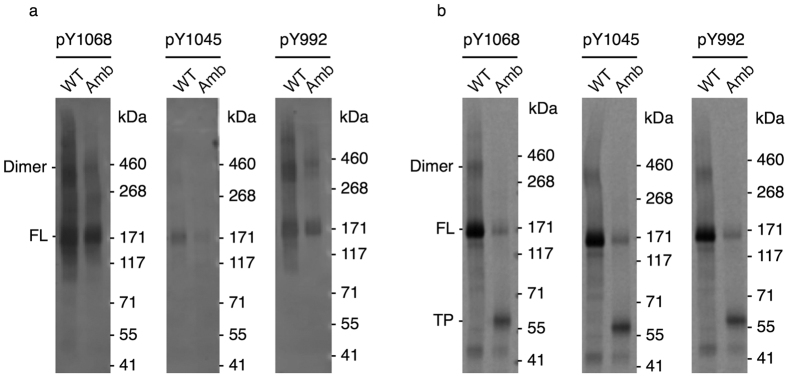
*In vitro* phosphorylation of selected tyrosine residues. (**a**) Immunoblotting of EGFR-eYFP (WT) and mutant with incorporated AzF (Amb) against phosphotyrosine 1068 (pY1068), 1045 (pY1045) and 992 (pY992) using specific antibodies. (**b**) Autoradiography of corresponding blotting membranes. Dimers as well as the full-length protein (FL) and the termination product (TP) are indicated. Blots (**a**) and autoradiograms (**b**) have been adapted in contrast, brightness and sharpness for better visibility. The original images can be found in supplementary Fig. 2.

Upon activation of the EGFR, its intrinsic receptor tyrosine kinase activity results in phosphorylation of several tyrosine residues within the C-terminal tail, which subsequently serve as recognition sites for adapter molecules thereby initiating different signal transduction pathways. Besides its ligand-induced activation, evidence has been provided indicating the capability of a ligand-independent mechanism, which seems to be promoted by high receptor densities within the membrane^25^. In order to verify the functionality of cell-free synthesized receptors we therefore performed a tyrosine kinase assay in the absence of ligand and analyzed phosphorylation of tyrosine residues 1068, 1045 and 992 by immunoblotting. Tyrosine residues 1068 and 992 both exhibited a high level of phosphorylation thereby underlining the functionality of cell-free synthesized wild type EGFR-eYFP and its corresponding amber mutant with AzF incorporated at position 687, whereas tyrosine 1045 was phosphorylated only to a minor extent (Fig. 3a). Autoradiography verified the presence of equal amounts of the receptors on the different blotting membranes (Fig. 3b). In addition to the full-length proteins (FL) and the termination product of the amber mutant (TP) another protein band was detected by autoradiography for the wild type on all blots approximately corresponding to the size of an EGFR-eYFP dimer. Although not as sharp, this dimer band was also visible on the immunoblots for tyrosine 1068 and 992 for both EGFR-eYFP variants but not for tyrosine 1045 (Fig. 3a). Estimation of the functional proportion of cell-free synthesized EGFR-eYFP was conducted in a semi-quantitative way after dephosphorylation followed by the kinase assay by immunoblotting with anti-EGFR and anti-phosphotyrosine 1068 antibodies (Supplementary Fig. 3) and revealed approximately 20% of receptors being phosphorylated in the absence of ligand (Supplementary Fig. 4). However, it is important to note that the results only reflect receptors activated in the absence of ligand. Thus, these results may represent only part of the actual fraction that is present in a functional form. Furthermore, the functionality of cell-free synthesized EGFR-eYFP and its corresponding amber mutant found in the supernatant fraction after fractionation of the reaction mixtures was analyzed and revealed no phosphorylation of tyrosine 1068 (Supplementary Fig. 5).

### *In vitro* interaction of cell-free synthesized EGFR with Grb2

**Figure 4:**
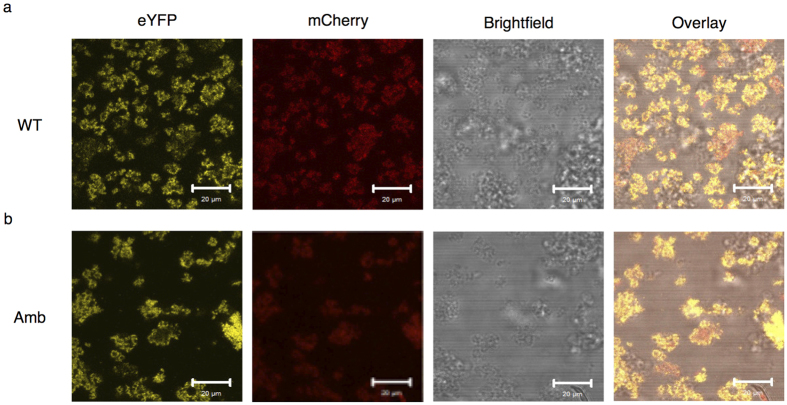
Interaction of cell-free synthesized receptors with Grb2-mCherry. Microsomal fractions containing EGFR-eYFP (WT (**a**)) and the mutant with incorporated AzF (Amb (**b**)) were incubated with soluble Grb2-mCherry in kinase buffer and after centrifugation subjected to CLSM.

Based on the finding that tyrosine 1068 is readily phosphorylated under the applied assay conditions and in its native context is known to be a docking site for the adapter protein Grb2^26^, the interaction of the two cell-free synthesized proteins was investigated by confocal laser scanning microscopy (CLSM) analysis. The highest yields of cell-free produced Grb2, synthesized as a mCherry fusion protein, were obtained by using the corresponding IRES template in the CECF reaction format with the majority of *de novo* synthesized protein being in a soluble form in the supernatant fraction (Supplementary Fig. 6a). Besides synthesis of the full-length Grb2-mCherry (53 kDa), autoradiography revealed synthesis of a set of different side products in the CECF format. The occurrence of side products in cell-free protein synthesis is not an uncommon observation, which can often be attributed to translation initiation events downstream of the first ATG codon or protein degradation by endogenous proteases that are not inhibited by the supplemented caspase inhibitor. Interestingly, IRES-mediated synthesis in the batch mode produced only the full-length Grb2-mCherry, indicating an effect of the prolonged reaction time on occurrence of the side products. Nevertheless, we functionalized microsomes with the wild type EGFR-eYFP as well as the AzF687 mutant and performed the tyrosine kinase assay in the presence of soluble cell-free synthesized Grb2-mCherry from the CECF reaction. Subsequently, we collected the microsomes by centrifugation and applied hypoosmotic conditions for CLSM analysis. As expected, localization of both receptor variants was found to be at the microsomal membranes rather than in their lumen or the surrounding environment, reflected by the occurrence of several fluorescent spheres (Fig. 4, eYFP). In accordance, Grb2-mCherry was likewise found at the microsomal membranes (Fig. 4, mCherry) and the overlay indicated co-localization of the receptor and adapter proteins (Fig. 4, Overlay). In contrast, no Grb2-mCherry was detected under the applied settings when microsomes from a control reaction without gene template were treated likewise (Supplementary Fig. 7).

**Figure 5:**
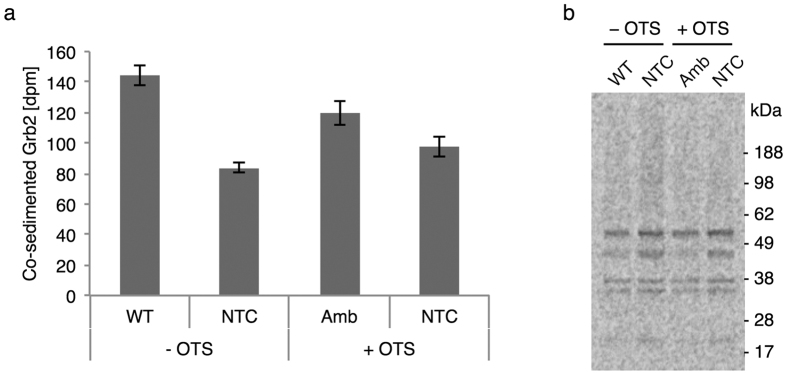
Co-sedimentation of hGrb2-mCherry with *Sf*21 microsomes containing cell-free synthesized receptors. (**a**) Radioactivity of isotopically labeled Grb2-mCherry co-sedimented with *Sf*21 microsomes containing the EGFR-eYFP (WT), the mutant with incorporated AzF (Amb) and no receptor (NTC). Cell-free reactions were carried out in the absence (−) and presence (+) of the orthogonal translation system (OTS). Error bars represent the standard deviation of triplicate analysis. (**b**) Corresponding autoradiograms of the supernatant fractions after co-sedimentation showing a decreased band intensity of Grb2-mCherry variants when incubated with microsomes containing receptor molecules.

To further verify interaction of the cell-free synthesized receptors and adapter molecules we performed a co-sedimentation assay with isotopically labeled Grb2-mCherry on microsomes containing either of the non-labeled receptor variants. β-scintillation counting verified the co-sedimentation of Grb2-mCherry when the microsomes contained one of the receptor variants thereby underlining their interaction *in vitro* (Fig. 5a). Furthermore, autoradiography of the supernatant from the co-sedimentation assay revealed that the predominant interaction was taking place with one of the Grb2-mCherry side products as the corresponding protein band migrating slightly lower than the full-length Grb2-mCherry almost disappeared when incubated with receptor-functionalized microsomes (Fig. 5b, WT and Amb).

## Discussion

Herein, we have demonstrated, that the combination of CrPV-IRES-mediated translation in a cell-free microsome-containing *Sf*21 system with prolonged reaction times, achieved by using the CECF reaction format, enabled increasing total protein yields of the human EGFR by more than 100-fold compared to the previously described standard conditions^17^. Thereby, several μg/ml *de novo* synthesized protein in the microsomal fraction originally obtained in the batch format were elevated to over 236 μg/ml within 24 hours and 285 μg/ml within 48 hours. This great synergistic effect is explained by addressing two major bottlenecks of cell-free protein synthesis based on eukaryotic cell extracts. On the one hand, the availability of translation initiation factors no longer limits overall protein synthesis using templates equipped with the CrPV-IRES and on the other hand, an early accumulation of byproduct as well as exhaustion of building blocks and energy equivalents is delayed by free diffusion between the reaction and the surrounding feeding compartment, thereby prolonging the reaction lifetime. Moreover, the prolonged incubation times revealed a positive effect on maturation of the eYFP moiety as the increase of fluorescence in the microsomal fraction was found to be more than 70-fold higher than the increase in total protein.

In comparison, the CECF reaction is approximately 10 times more expensive than the batch reaction due to the necessity of supplementing the 1 ml feeding mixture with building blocks and energy equivalents. As the combination of IRES template and CECF mode resulted in a more than 100-fold increase of total EGFR-eYFP after 24 hours, it can be concluded that for the same price at least 10 times more total protein can be obtained at the cost of a 22 hours elevated time effort. The labor effort is basically the same for both reaction formats. Based on the underlying costs for materials used in this work, 1 μg of EGFR-eYFP can be produced for 1.6 USD or 1.4 Euro by a 24 hour synthesis using the IRES-template in the CECF reaction format.

Incorporation of the non-canonical amino acid AzF by amber suppression using an orthogonal tRNA/synthetase pair together with the novel synthesis conditions was found to occur with an efficiency of only 7% compared to the synthesis of the wild type protein, but estimation of full-length suppression product yields revealed a total of approximately 17 μg/ml. In contrast, utilization of the OcfTS under standard conditions without additional supplementation of Mg^2+^, which was previously found to enhance amber suppression using the presented OcfTS^17^, did not provide any detectable amounts of the full-length suppression product.

It has previously been demonstrated that the microsome-containing cell-free *Sf*21 system is capable of providing proteins including the EGFR with N-linked glycans^17^,^18^,^19^. Thus the faint bands migrating slightly above the calculated molecular weight of the EGFR-eYFP and its amber variant in the microsomal fractions can be assigned the glycosylated receptors. The fact that the glycosylated receptors are no longer visible after 24 and 48 hours can be explained by the limited availability of glycan building blocks that is exhausted over time and cannot account for the high amount of cell-free synthesized protein when combining the IRES-mediated synthesis with the CECF reaction format. The signal of the comparatively low amount of glycosylated receptors is therefore not distinguishable anymore. Most likely, providing additional glycan building blocks can enhance the glycosylation capacity of the system. As so far no indication for Golgi-mediated glycosylation has been found in the cell-free *Sf*21 system, the glycan composition must be limited to core glycosylation taking place in the ER^27^.

Taking into account that the EGFR is a high molecular weight integral membrane protein of human origin, the presented yields can be considered highly competitive with other cell-free systems such as from *E. coli* or wheat germ embryos^28^,^29^. In particular, the major benefit of the presented *Sf*21 system is its inherent eukaryotic translation and folding machinery in combination with the translocationally-active, ER-derived microsomes, providing an excellent environment to support the production of properly folded, membrane-embedded and functional proteins as demonstrated by assessment of the phosphorylation activity of cell-free synthesized EGFR-eYFP variants. On top, the *Sf*21 microsomes functionalized with receptor molecules represent an ideal platform for structural and functional *in vitro* studies as exemplified by investigation of the EGFR/Grb2 interaction. For instance, the observation that tyrosine 1045, which is known to provide a negative regulation of ligand induced receptor tyrosine kinase signaling by ubiquitination and subsequent receptor degradation^30^, is phosphorylated only to a minor extent may imply that this mechanism of EGFR “downregulation” is less important in the context of ligand independent receptor activation. In this context, biological investigations can be further assisted by incorporation of non-canonical amino acids with desired characteristics such as the photosensitivity^31^ and selective chemical reactivity^32^ of the azide using the presented OcfTS.

## Methods

### Template generation and site-directed mutagenesis

Templates harboring the human EGFR gene with its native signal sequence substituted by the melittin signal sequence (Mel) and fused to eYFP (pIX3.0-Mel-EGFR-eYFP) and the mutant *E. coli* tyrosyl-tRNA synthetase genes AzFRS (Thr37, Ser182, Ala183; pXAzFRS-SII) and eAzFRS (Thr37, Ser182, Ala183, Arg265; pQE2-eAzFRS-SII) both followed by a StrepTag II (SII; IBA) were generated as described previously^17^. All plasmids used herein contain the T7 promoter and terminator sequences upstream and downstream of the ORFs, respectively. To introduce the CrPV-IRES the corresponding region was excised from a pIX3.0-CrPV-Mel-eYFP vector using NotI and BstZ17. The pIX3.0-Mel-EGFR-eYFP was treated likewise and dephosphorylated using CIP (calf intestinal alkaline phosphatase, NEB) to prevent religation of the linearized plasmid. Finally, the CrPV insert was ligated into the linearized vector pIX3.0-Mel-EGFR-eYFP to obtain pIX3.0-CrPV-EGFR-eYFP. The amber stop codon at position 687 was introduced into the EGFR gene by PCR using mismatch primer pairs. The integrity of all constructs was verified by sequencing. Plasmid preparations suitable for cell-free protein synthesis were carried out using JETSTAR Plasmid Purification Kits (GENOMED) starting from transformed *E. coli* XL10-Gold ultracompetent cells (Agilent).

### Cell-free protein synthesis and incorporation of AzF

Preparation of the microsome-containing *Sf*21 extract as well as the coupled cell-free protein synthesis have been described previously^17^,^19^. In brief, cell-free reactions from templates without the CrPV-IRES were constituted using three different stable premixes stored at −80 °C. Premix A (10x) was composed of 300 mM HEPES-KOH (pH 7.6), 750 mM KOAc, 2.5 mM spermidine, 1 mM of the 20 standard amino acids each (Merck) and 29 mM Mg(OAc)_2_. Premix B (2.5x) contained the S7 nuclease-treated *Sf*21 extract supplemented with 250 μg/ml creatine kinase (Roche) and 50 μg/ml bulk yeast tRNA (Roche). Premix C (5x) consisted of 100 mM creatine phosphate, 8.75 mM ATP, 1.5 mM CTP, 1.5 mM UTP, 1.5 mM GTP (Roche) and 1.65 mM m7G(ppp)G cap analogue (Prof. Edward Darzynkiewicz, Warsaw University, Poland). First, the volume of additional RNase-free water necessary to sum up to the final reaction volume of 50 μl was calculated and pipetted into a 1.5 ml Eppendorf tube. Next, 5 μl of premix A and 20 μl of premix B were added. Then, the caspase inhibitor Z-VAD-FMK (benzyloxycarbonyl-Val-Ala-Asp(OMe)-fluoromethylketone, Promega) was supplemented at a final concentration of 30 μM, sodium azide at 0.02% and T7 RNA polymerase (Agilent) was added to result in 1 U/μl. Finally, 10 μl premix C were added and the protein synthesis reaction was initiated by addition of template DNA at a final concentration of 60 ng/μl. Isotopic labeling was achieved by supplementation of ^14^C-leucine at 20 μM (specific radioactivity 25 dpm/pmol, Perkin Elmer). It should be noted that all components and premixes were thawed on ice, gently mixed and then stored on ice during the time of constituting the cell-free reactions. Moreover, after addition of each component, the reaction solution was gently mixed by slowly pipetting up and down and after addition of the template DNA the reaction mixture was gently mixed and spun down at up to 800 × g. Incubation was carried out at 27 °C and gentle shaking for 90–120 minutes (Thermomixer comfort, Eppendorf). Cell-free reactions from templates harboring the CrPV-IRES were constituted likewise but with a slightly different version of premix A containing 1500 mM KOAc and 39 mM Mg(OAc)_2_. The CECF reactions were constituted likewise but incubation was carried out in commercially available two-chamber dialysis devices composed of a 50 μl reaction and a 1000 μl feeding compartment. The feeding mix contained all the components of the reaction mix except for premix B and T7 RNA polymerase.

Preparation of the mutant tyrosyl-tRNA synthetases was carried out as previously described^17^,^22^. *In vitro* transcribed suppressor tRNA_CUA_ was purchased from RiNA (RiNA GmbH, Berlin, Germany). Site-directed incorporation of AzF was achieved by supplementation of cell-free reaction mixtures with 2 μM eAzFRS, 2 μM tRNA_CUA_ and 2 mM AzF (Bachem). The feeding mix for the CECF reactions was only supplemented with AzF. Incubation was performed in the dark as described above.

### Determination of total protein yields and fluorescence detection of fusion proteins

Following cell-free protein synthesis in the presence of ^14^C-leucine, reactions were fractionated into the supernatant and the microsomal fraction by centrifugation at 16.000 × g and 4 °C for 10 minutes. The microsomal fractions were resuspended in equal volumes of PBS. Subsequently, aliquots of 5 μl (from templates without IRES) or 2.5 μl (from templates with IRES) were subjected to hot trichloroacetic acid precipitation and liquid scintillation counting in triplicates as described previously^17^. Total protein yields were calculated from measured disintegrations per minute (dpm) taking into account the specific radioactivity, the molecular mass and the number of leucines of the synthesized proteins.

Fluorescence of the fusion proteins was measured from 5 μl aliquots of the corresponding fractions in 95 μl PBS solution on black 96 well microplates (Berthold) using the “Mithras^2^ LB 943 Monochromator Multimode Reader” (Berthold). For eYFP detection, samples were excited at 485 nm and emission was detected at 530 nm. For mCherry detection samples were excited at 540 nm and emission was detected at 590 nm. The background fluorescence of control measurements from cell-free reactions carried out under identical conditions but in the absence of a gene template was subtracted from the measured intensities.

### Tyrosine kinase assay and Grb2 binding

To allow for *in vitro* autophosphorylation of receptors embedded in the *Sf*21 microsomal membranes, microsomal fractions from 10 μl of the complete reaction mixture were collected and resuspended in 20 μl kinase buffer composed of 100 mM HEPES (pH 7.4), 1% glycerol, 0.1 mg/ml BSA, 5 mM MgCl_2_, 1.25 mM MnCl_2_, 0.1 mM NaVO_3_, 2 μM caspase inhibitor and 200 μM ATP or RNase-free water. Incubation was carried out for 30 minutes at room temperature.

Grb2 binding was achieved by performance of the tyrosine kinase assay as described above but including 2 μl of soluble Grb2-mCherry from the supernatant of the corresponding CECF reaction. Subsequently, the samples were centrifuged to collect the microsomes and confocal images were taken under hypoosmotic conditions on a LSM 510 meta (Zeiss) laser-scanning microscope. Two channels were used to detect eYFP (excitation 514 nm, meta detector >529 nm) and mCherry (excitation 543 nm, meta detector >593 nm).

In addition, Grb2 binding was carried out with isotopically labeled Grb2-mCherry. Following the binding assay, samples were centrifuged to separate the soluble fraction from the microsomal fraction. The microsomal fractions were analyzed quantitatively as described above and the supernatant fractions were subjected to electrophoretic separation and autoradiography as described below.

### Denaturing PAGE, in-gel fluorescence, immunoblotting and autoradiography

Sample preparation including cold acetone precipitation followed by denaturing PAGE using NuPAGE 10% Bis-Tris and 3–8% Tris-Acetate precast gels (Life Technologies) as well as autoradiography were performed as described previously and in accordance to the manufacturer’s instructions^17^. Due to increased protein concentrations resulting from utilization of the CrPV-IRES and the CECF reaction format, the amount of sample applied to the denaturing PAGE was reduced to correspond to 2.5 μl of the initial fractions from the cell-free reactions. For reactions were the wild type EGFR-eYFP was synthesized with IRES in the CECF format for 24 and 48 hours, the sample was further reduced to correspond to 0.25 μl of the initial fractions.

Immunoblotting was performed using the “IBlot Gel Transfer Device” (Life Technologies) according to the manufacturer’s instructions. Following denaturing PAGE (3–8% Tris-Acetate) proteins were transferred to a PVDF membrane (Life Technologies). The membrane was blocked in “Roti-Block” (Roth) for 4 hours and subsequently incubated with “Phospho-EGF Receptor (Tyr1068) (D7A5) XP^®^ Rabbit mAb 3777”, “Phospho-EGF Receptor (Tyr1045) XP^®^ Rabbit pAb 2237” or “Phospho-EGF Receptor (Tyr992) XP^®^ Rabbit pAb 2235” primary antibodies diluted 1:1000 over night at 4 °C. “Anti-rabbit IgG, HRP-linked Antibody 7074” diluted 1:2000 was used as a secondary antibody and detection was carried out using the “Amersham ECL Prime Western Blotting Detection Reagent” (GE Healthcare) and the “Typhoon Trio+ Variable Mode Imager” (GE Healthcare). After detection blotting membranes were dried and subjected to autoradiography.

## Additional Information

**How to cite this article**: Quast, R. B. *et al*. High-yield cell-free synthesis of human EGFR by IRES-mediated protein translation in a continuous exchange cell-free reaction format. *Sci. Rep.* **6**, 30399; doi: 10.1038/srep30399 (2016).

## Supplementary Material

Supplementary Information

## Figures and Tables

**Table 1:** Total protein yields of wild type EGFR-eYFP obtained using the corresponding standard (−) and IRES template (+) in the batch and CECF format.

Fraction	Mode	Time	IRES	Total Protein [μg/ml]
Supernatant fraction	Batch	2 h	−	1.8 ± 0.3
		2 h	+	5.9 ± 0.4
	CECF	24 h	−	8.5 ± 0.4
		24 h	+	57 ± 1
		48 h	+	43 ± 6
Microsomal fraction	Batch	2 h	−	2.2 ± 0.1
		2 h	+	8.5 ± 0.8
	CECF	24 h	−	8.4 ± 0.1
		24 h	+	237 ± 6
		48 h	+	286 ± 21

Errors represent the standard deviation of triplicate analysis.
