# Supplementary material for: High-yield cell-free synthesis of human EGFR by IRES-mediated protein translation in a continuous exchange cell-free reaction format
Source: Sci Rep. 2016 Jul 26;6:30399. doi: 10.1038/srep30399 (PMC4960648; doi:10.1038/srep30399)
Supplement: Supplementary Information [file srep30399-s1.pdf]

Supplementary Information

High-yield cell-free synthesis of human EGFR by IRES-mediated protein translation in a continuous exchange cell-free reaction format

Authors

Robert B. Quast, Andrei Sonnabend, Marlitt Stech, Doreen A. Wüstenhagen, Stefan Kubick

Affiliations

Fraunhofer Institute for Cell Therapy and Immunology (IZI), Branch Bioanalytics and Bioprocesses (IZI-BB), Am Mühlenberg 13, D-14476 Potsdam, Germany

To whom correspondence should be addressed: Dr. Stefan Kubick, Fraunhofer Institute for Cell Therapy and Immunology (IZI), Branch Bioanalytics and Bioprocesses (IZI-BB), Am Mühlenberg 13, 14476 Potsdam, stefan.kubick@izi-bb.fraunhofer.de, Tel: +49 331 58 187 -306 / Fax -119

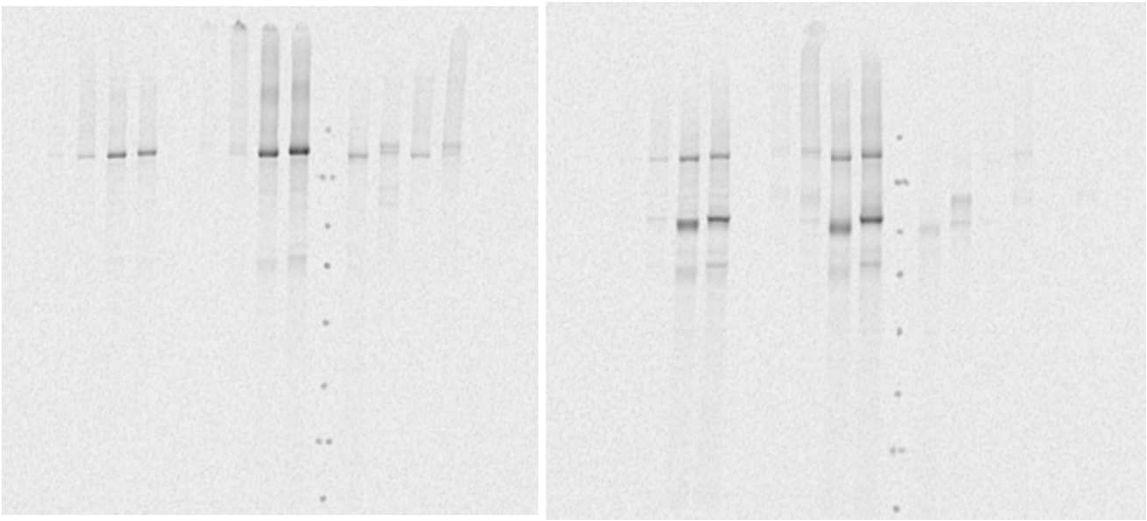

Supplementary figure 1 | Original unmodified autoradiograms shown in figure 1 (left) and 2 (right).

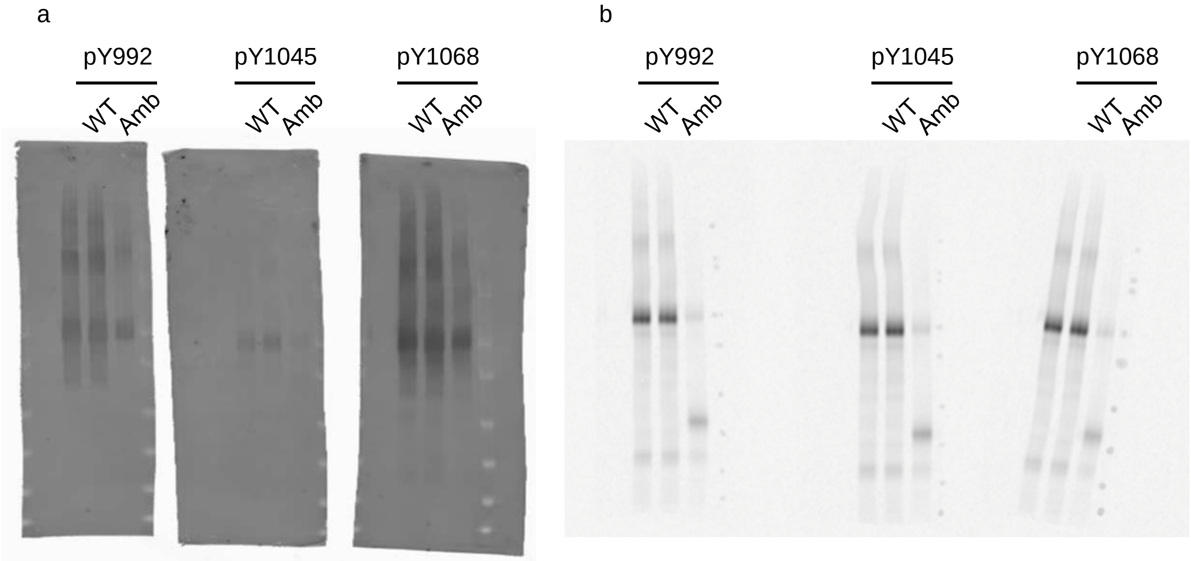

Supplementary figure 2 | Original unmodified blots (a) and corresponding autoradiograms (b) shown in figure 3. Unspecified lanes have no relevance in this publication.

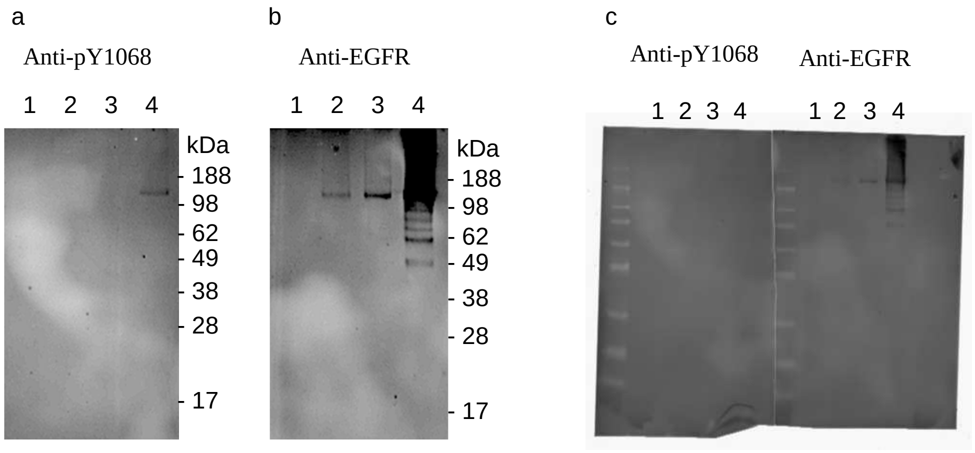

Supplementary figure 3 | Comparison of total and functional amount of EGFR-eYFP in the microsomal fraction. Aliquots of the microsomal fraction of cell-free synthesis reactions using the CrPV-IRES-EGFR-eYFP template in the CECF mode were either used directly for electrophoretic separation followed by immunoblotting (2), preliminarily dephosphorylated in 1 x cut smart buffer with calf intestinal phosphatase (3) or dephosphorylated and subsequently incubated in kinas buffer with ATP (4). A control reaction without a gene template (1) was performed in parallel. The phosphorylated receptors were detected using an EGFR specific antibody (b) and a phosphotyrosine 1068 specific antibody (a). The contrast, brightness and sharpness have been adapted in a and b. c shows the original non-adapted blots.

a

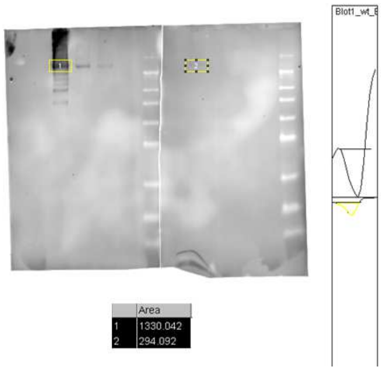

b

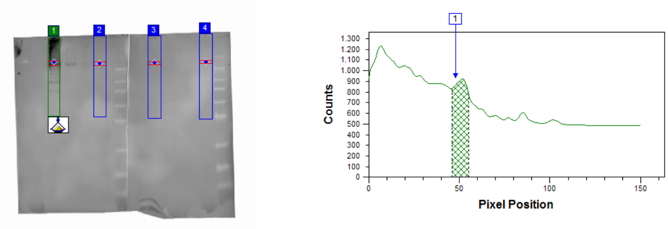

	Band No	Position	Volume	Vol+BkGnd	Calib Vol(ng)	Area	Band %	Band % (Norm)	Lane %	MW (kd)	Rf
Lane 1	1	48	174244,76	174244,76	-	198,00	100,00	0,00	7,56	**188,000**	0,320
Lane 2	1	52	79307,81	79307,81	-	144,00	100,00	0,00	3,94	-	0,338
Lane 3	1	51	85367,89	85367,89	-	144,00	100,00	0,00	4,11	-	0,327
Lane 4	1	54	66821,69	66821,69	-	144,00	100,00	0,00	3,60	-	0,333

Supplementary figure 4 | Screenshots of estimation of functional proportion of EGFR-eYFP based on western blots shown in supplementary figure 3 using either the ImageJ (a) or the ImageQuant (b) software.

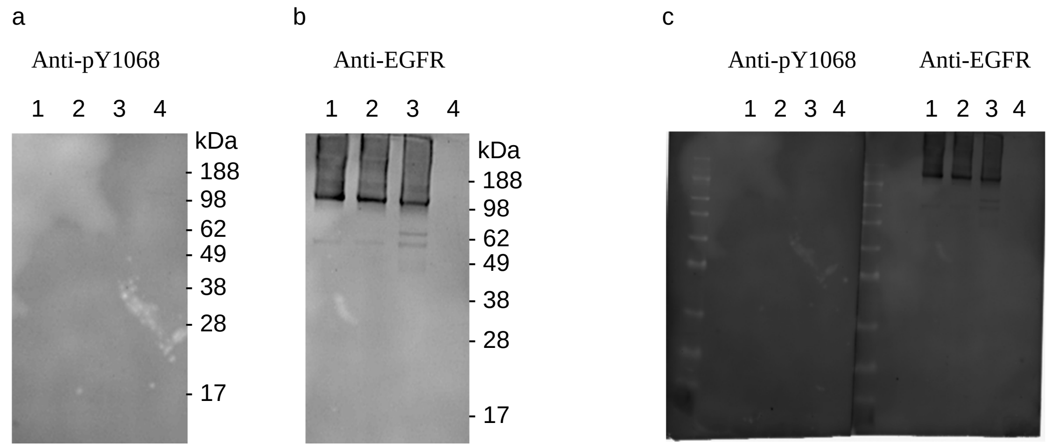

Supplementary figure 5 | Comparison of total and functional amount EGFR-eYFP in the supernatant fraction. Aliquots of the supernatant fraction of cell-free synthesis reactions using the CrPV-IRES-EGFR-eYFP template in the CECF mode were either used directly for electrophoretic separation followed by immunoblotting (1), preliminarily dephosphorylated in 1 x cut smart buffer with calf intestinal phosphatase (2) or dephosphorylated and subsequently incubated in kinase buffer with ATP (3). A control reaction without a gene template (4) was performed in parallel. The phosphorylated receptors were detected using an EGFR specific antibody (b) and a phosphotyrosine 1068 specific antibody (a). The contrast, brightness and sharpness have been adapted in a and b. c shows the original non-adapted blots.

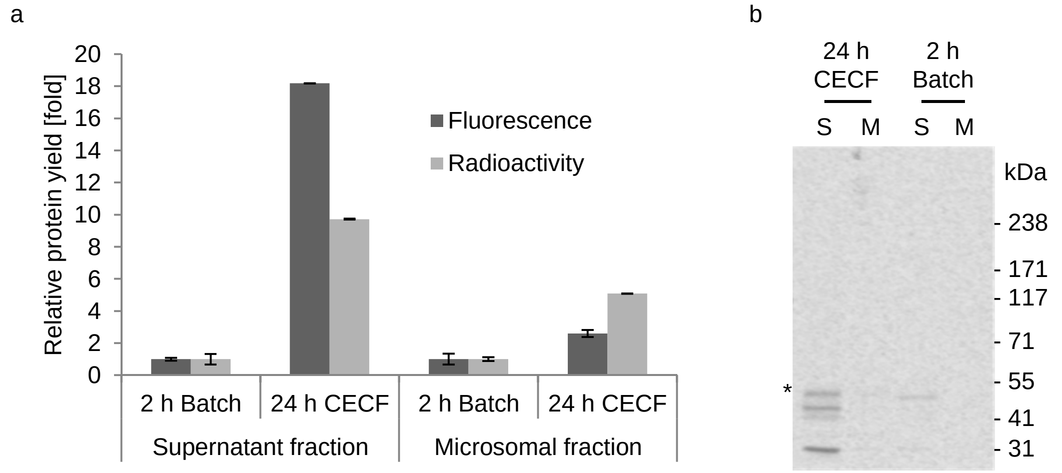

Supplementary figure 6 | IRES-mediated cell-free synthesis of Grb2-mCherry in batch and CECF reaction mode. a) Relative protein yields in supernatant (SF) and microsomal fraction (MF) in relation to the standard batch reaction (+ IRES) determined by analysis of mCherry fluorescence and incorporated ^{14}C-leucine. Error bars represent the standard deviation of triplicate analysis. b) Corresponding autoradiogram after electrophoretic protein separation. The full-length Grb2-mCherry is marked by an asterisk.

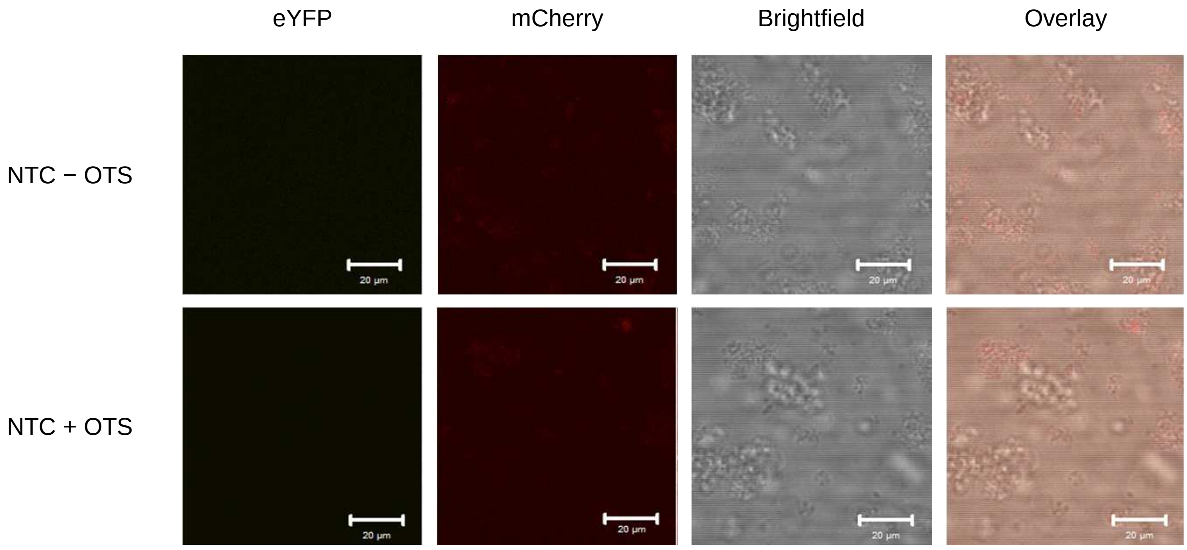

Supplementary figure 7 | Interaction of cell-free synthesized Grb2-mCherry with *Sf*21 microsomes in the absence of receptor. Microsomal fractions from control reactions without gene template (NTC) carried out in the absence (− OTS) or presence of the orthogonal translation system (+ OTS) were incubated with soluble Grb2-mCherry in kinase buffer and after centrifugation subjected to CLSM.
